# Work-Related Fear-Avoidance Beliefs and Risk of Low-Back Pain: Prospective Cohort Study Among Healthcare Workers

**DOI:** 10.1007/s10926-024-10221-y

**Published:** 2024-08-05

**Authors:** Markus Due Jakobsen, Jonas Vinstrup, Lars Louis Andersen

**Affiliations:** 1 National Research Centre for the Working Environment, Lersø Parkalle 105, Copenhagen, Denmark; 2 Department of Health Science and Technology, Aalborg University, Aalborg, Denmark

**Keywords:** Musculoskeletal pain, Occupational health, Fear avoidance, Catastrophizing, Nursing, Pain management

## Abstract

**Purpose:**

Low-back pain (LBP) is a prevalent condition among healthcare workers, negatively affecting well-being and work ability. Research has identified fear-avoidance beliefs, i.e., the belief that physical activities worsen or prolong pain, as a key psychological factor in LBP. Given the physical demands of healthcare work, understanding the link between fear-avoidance and LBP is crucial for effective prevention and management strategies. This study investigated the prospective association between fear-avoidance beliefs and risk of increased LBP intensity and duration in hospital workers.

**Methods:**

Fear-avoidance beliefs and LBP were assessed in 1933 healthcare workers from 389 departments at 19 hospitals at baseline and 1-year follow-up. Associations between baseline work-related fear-avoidance beliefs (FABW) and LBP intensity and duration at follow-up were analyzed using cumulative logistic regression, adjusting for various factors including age, sex, baseline LBP, education, seniority, patient transfers, psychosocial work environment, and lifestyle.

**Results:**

Moderate and high FABW was associated with higher odds of increased pain intensity (OR: 1.37 [95% CI 1.09–1.73] and 1.85 [95% CI 1.18–2.88], respectively) and prolonged pain duration (OR: 1.37 [95% CI 1.05—1.78] and 2.27 [95% CI 1.50–3.44], respectively). A sensitivity analysis including only female nurses showed similar results, with the high FABW group having significantly higher odds of increased pain intensity (OR 2.95, 95% CI 1.84–4.72) and duration (OR 2.64, 95% CI 1.55–4.49).

**Conclusions:**

Fear-avoidance beliefs increase the risk of LBP intensity and duration among healthcare workers, emphasizing the need for interventions dealing with psychological aspects of LBP.

## Introduction

The global shortage of the healthcare workforce can be attributed to multiple factors [1]. However, the character of the work and its associated health-related consequences stand out as significant driving forces [2, 3]**.** Healthcare work is physically strenuous, involving frequent exposure to sudden and high loadings of the spine during patient transfer, often requiring twisting and bending of the back [4, 5]. This demanding nature of healthcare work increase the risk of low-back pain (LBP) [6–10] s. 2, [11], long-term sickness absence [12, 13] and disability pension [14–16]. Additionally, healthcare workers face psychosocial stressors, including long work hours, high workloads, and emotional strain, which further exacerbate the development and progression of LBP [2, 17, 18]. Thus, understanding the factors contributing to LBP in healthcare workers is crucial for addressing the challenges posed by the global healthcare workforce shortage.

Fear-avoidance behavior (FAB) is a psychological phenomenon characterized by the avoidance of movements or activities due to fear of increased pain or re-injury [19]. In the development of chronic LBP, psychological factors play a significant role, which is illustrated by the widely accepted fear-avoidance model [20]. According to this model, negative beliefs about pain and illness can initiate a cycle of fear and avoidance, where individuals interpret activity and movement as potentially harmful or painful, resulting in avoidance behaviors. This avoidance behavior perpetuates negative beliefs, leading to a detrimental cycle of reduced activity and increased distress [20]. Individuals without fear-avoidance beliefs are more likely to confront pain problems and actively engage in coping strategies, making interventions targeting high fear-avoidance beliefs focus on promoting positive coping mechanisms [20]. Consequently, fear-avoidance has significant implications for the experience and management of LBP.

Previous research has extensively explored the association between general fear-avoidance and LBP in various populations, revealing its impact on sickness absence, disability, recovery duration, and treatment outcomes in individuals with LBP [21–25]. Fear-avoidance beliefs are considered mediators between pain and avoidance behaviors like sick leave or early retirement [26–29]. However, limited research focuses on the relationship between work-specific fear-avoidance behavior and LBP among healthcare workers [25, 30].

Fear-avoidance beliefs are commonly assessed through the two-part Fear-Avoidance Beliefs Questionnaire (FABQ), which distinguishes between fear avoidance related to general physical activities (FABP) and those specific to work (FABW) [19]. This distinction underscores the importance of investigating work-related fear-avoidance beliefs among healthcare professionals. Understanding this relationship becomes pivotal for designing targeted interventions and preventive measures that address their distinctive challenges, encompassing physically demanding tasks and psychosocial stressors. Such insights can contribute significantly to the effective management of LBP in this occupational group. By addressing fear-avoidance beliefs early on, healthcare workers can effectively manage pain, maintain productivity, and prevent negative consequences associated with chronic LBP, ultimately enhancing the quality of care provided, work ability, and overall well-being. Consequently, this study aims to examine the impact of work-related fear-avoidance beliefs on the intensity and duration of low-back pain among healthcare workers, irrespective of their initial pain status. Specifically, it seeks to understand how fear-avoidance beliefs related to work influence the progression or alleviation of LBP, thereby providing insights into both the development and exacerbation of LBP within this occupational group.

## Methods

### Study Design and Participants

This prospective study utilizes baseline and 1-year follow-up questionnaires, gathered from spring 2017 to 2018, to examine the associations between fear-avoidance levels and the intensity and duration of LBP. Specifically, the study investigates potential dose–response relationships between fear-avoidance measured subjectively at baseline, using fear-avoidance beliefs about work (FAB-work), and the likelihood of experiencing higher values on the pain intensity and pain duration scales, respectively.

7025 hospital workers across 389 departments at 19 hospitals in Denmark were invited to participate in the study. The included participants were healthcare workers who frequently engage with patients, including nurses, healthcare assistants, nurses aids, physical and occupational therapists, medical doctors, midwives, porters, and radiographers, regardless of their baseline pain status. To be included in the analysis, participants needed to respond to both the baseline and follow-up questionnaires, which consisted of the same questions. There were no specific exclusion criteria based on the presence or absence of low-back pain at baseline to allow for a comprehensive analysis of fear-avoidance beliefs among healthcare workers. The reporting of the manuscript adheres to the STROBE guidelines [31].

### Outcome Variables

#### LBP Intensity

Intensity of LBP was measured using the following question: *“Rate your pain for the low-back within the previous 4 weeks (0–10, where 0 is no pain and 10 is worst imaginable pain)”.*

#### LBP Duration

Duration of LBP was assessed with the question: *“How long have you experienced pain in the lower back (think back to when it started)?”* The response options included: “Not at all”, “ < 3 months”, “3–6 months”, “6–12 months”, and “ > 1 year”.

### Determinant

#### Fear-Avoidance Beliefs

Fear-avoidance beliefs were assessed using the fear-avoidance beliefs questionnaire (FABQ) developed by Waddell et al. [19]. The FABQ consists of two subscales: fear-avoidance beliefs about work (FABW) and fear-avoidance beliefs about physical activity. These scales measure individuals’ beliefs regarding how work and physical activity influence pain and the extent to which these activities should be avoided. A shortened version of the FABQ, only comprising work-related aspects (items 6–16) from the original questionnaire, was used to measure fear-avoidance behavior during work (FAB-work). Each item was rated on a scale of 0 to 6: 0 for disagreement, 3 for uncertainty, and 6 for agreement. An example item from the FABW scale is “*I should not do my normal work with my present pain*”. To prepare data for analysis, the responses were averaged and standardized on a scale of 0 to 42, where a higher score indicates a greater level of disagreement. Subsequently, the scores were divided into three categories based on the following score ranges: (1) “low fear-avoidance” (0 to 19), (2) “moderate fear-avoidance” (20 to 29) and (3) “high fear-avoidance” (30 to 42) [23, 32].

## Covariates

But the analysis accounts for a range of potential confounding factors that could influence pain intensity and development of pain, including individual characteristics, psychosocial environmental conditions, and workplace factors. These factors include age, sex, LBP at baseline, education, body mass index, smoking status, seniority, physical activity during leisure time, frequency of patient transfer, as well as factors related to the psychosocial work environment, such as recognition and influence at work [16, 33, 34].

## Ethics

In accordance with the Danish Data Protection Agency, the National Research Centre for the Working Environment registered all questionnaire studies internally. As per Danish law, informed consent and approval from ethical and scientific committees were not required for questionnaire- and register-based studies. Data were de-identified and analyzed anonymously to ensure confidentiality.

## Statistics

Cumulative logistic regression (Proc Genmod, SAS) was employed to assess the associations between fear-avoidance beliefs at work (FABW) at baseline and subsequent changes in low-back pain (LBP) intensity and duration at follow-up. The odds ratios (OR) derived from this analysis quantify the likelihood of experiencing higher values on the pain intensity and duration scales, demonstrating changes in these outcomes irrespective of initial pain levels. This approach allows us to understand how baseline FABW influences the progression or alleviation of LBP over the study period. The statistical analyses were adjusted for the covariates. The minimally adjusted analysis accounted for age, sex, and baseline LBP, while the fully adjusted analysis incorporated additional factors including age, sex, baseline LBP, education level, body mass index (BMI), smoking status, years of service, leisure-time physical activity, patient transfer frequency, and psychosocial workplace factors such as job recognition and influence. To address potential clustering effects, the hospital department variable was included in the “repeated subject” statement. The estimates are presented as ORs with corresponding 95% confidence intervals. Moreover, sensitivity analyses were conducted in a similar manner to investigate the associations within the subgroup of female nurses specifically.

## Results

**Fig. 1:**
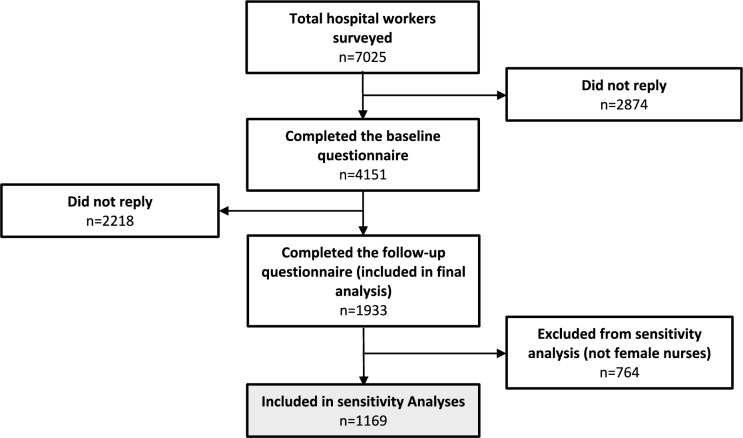
Flow of study participants. Participants included in the sensitivity analysis are marked with grey

Out of the 7025 hospital workers from 389 departments at 19 hospitals in Denmark who were invited to participate, 4151 (59%) completed the full baseline questionnaire. Of those, a total of 1933 healthcare workers completed both the baseline and follow-up questionnaires. Following the initial analysis, sensitivity analyses were carried out to investigate associations within a distinct subgroup—female nurses, who numbered 1169. The baseline characteristics of these participants are detailed in Table 1 and the flow of participants is depicted in Fig. 1.

**Table 1:** Baseline characteristics, health, and psychosocial work factors of healthcare workers

Variable	Description	All participants (*N* = 1933)	Female nurses (*N* = 1169)
*FAB Work Subscale*			
Normal FABW (0–19)	%	81.17% (1569)	80.67% (943)
Moderate FABW (20–29)	%	15.00% (290)	15.57% (182)
High FABW (30–42)	%	3.83% (74)	3.76% (44)
*Demographics*			
Gender (% women)		86.82% (1732)	100% (1195)*
Age (years)	Mean (SD)	48.82 (10.68)	48.36 (10.40)
*Work-Related*			
Years in Profession	Mean (SD)	18.32 (11.56)	18.53 (11.37)
Daily Patient Transfers	Mean (SD)	3.51 (2.18)	3.81 (2.09)
*Psychosocial Work Factors (0–100, where 100 is best)*			
Collaboration and Support from Colleagues	Mean (SD)	78.44 (16.62)	78.98 (15.90)
Influence at Work	Mean (SD)	73.74 (17.49)	73.92 (17.08)
*Health Factors*			
Low-back Pain Intensity (0–10)	Mean (SD)	2.31 (2.52)	2.33 (2.49)
*Lifestyle Factors*			
Smoking (% Yes)	%	Yes: 8.42%, No: 91.58%	Yes: 7.95%, No: 92.05%
BMI (mean)	Mean (SD)	25.22 (4.56)	25.21 (4.66)
Leisure Physical Activity		Sedentary: 6.68%, Light Activities ≥ 4 h/week: 62.60%, Physical Exercise ≥ 4 h/week: 27.66%, Hard Exercise & Competitions Regularly: 3.06%	Sedentary: 5.36%, Light Activities ≥ 4 h/week: 64.85%, Physical Exercise ≥ 4 h/week: 27.87%, Hard Exercise & Competitions Regularly: 1.92%

As depicted in Tables 2 and 3, the analyses reveals that moderate to high fear-avoidance beliefs about work are associated with elevated odds of increased pain intensity and pain duration at the one-year follow-up. Among the overall population, individuals with high levels of fear-avoidance had higher odds of increased pain intensity at one-year follow-up compared to those with low fear-avoidance (OR: 1.85, 95% CI 1.18–2.88, *p* = 0.0069) (Table 2). Similarly, individuals with moderate fear-avoidance levels had higher odds of increased pain intensity (OR: 1.37, 95% CI 1.09–1.73, *p* = 0.0072) compared to those with low fear-avoidance.

**Table 2:** Odds ratios (OR) representing the association between fear-avoidance beliefs about work (FABW) at baseline and the likelihood of increased pain intensity at one-year follow-up among the entire study population and in the sensitivity analyses among female nurses

Fear-avoidance	All—minimally adjusted		All—fully adjusted	
	OR	95% CI	OR	95% CI
Moderate vs. Low	1.52	1.21—1.91	1.37	1.09—1.73
High vs. Low	2.11	1.38—3.22	1.85	1.18—2.88

	Nurses—Minimally adjusted		Nurses—Fully adjusted	
	OR	95% CI	OR	95% CI
Moderate vs. Low	1.26	0.97—1.63	1.16	0.89—1.52
High vs. Low	3.01	1.92—4.69	2.95	1.84—4.72

*The minimally adjusted model accounts for age, sex, and initial pain levels, while the fully adjusted model also considers education, BMI, smoking, work experience, leisure activity, patient handling frequency, and psychosocial work factors

**Table 3:** Odds ratios (OR) representing the association between fear-avoidance beliefs about work (FABW) at baseline and the likelihood of increased pain duration at one-year follow-up among the entire study population and in the sensitivity analyses among female nurses

Fear-avoidance	All—minimally adjusted		All—fully adjusted	
	OR	95% CI	OR	95% CI
Moderate vs Low	1.43	1.11—1.84	1.37	1.05—1.78
High vs. Low	2.29	1.52—3.44	2.27	1.50—3.44

	Nurses—minimally adjusted		Nurses—fully adjusted	
	OR	95% CI	OR	95% CI
Moderate vs Low	1.54	1.10—2.17	1.44	1.02—2.05
High vs Low	2.61	1.54—4.43	2.64	1.55—4.49

*The minimally adjusted model accounts for age, sex, and initial pain levels, while the fully adjusted model also considers education, BMI, smoking, work experience, leisure activity, patient handling frequency, and psychosocial work factors

Regarding pain duration, the analysis revealed that individuals experiencing high fear-avoidance had 2.27 times higher odds of prolonged pain duration (95% CI 1.50–3.44, *p* = 0.0001), while those with moderate fear-avoidance levels had 1.37 times higher odds of prolonged pain duration (95% CI 1.05–1.78, *p* = 0.02) (Table 3).

The sensitivity analysis among female nurses further supported these findings, with the high fear-avoidance group consistently showing significantly higher odds of increased pain intensity (OR 2.95, 95% CI 1.84–4.72, *p* < 0.0001) and higher odds of increased pain duration (OR 2.64, 95% CI 1.55–4.49, *p* = 0.0003). Although the moderate fear-avoidance group did not reach statistical significance for pain intensity (OR 1.16, 95% *CI* 0.89–1.52, *p* = 0.2742), it remained significant for pain duration (OR 1.44, 95% CI 1.02–2.05, *p* = 0.0398).

Table 4 illustrates the one-year follow-up of low-back pain intensity across the general study population and specifically among female nurses, segmented by fear-avoidance belief (FABW) categories. The data reveal a consistent pattern: as FABW levels escalate from normal to high, the reported pain intensity increases in both the entire study cohort and the nurse subgroup, underscoring the direct relationship between fear-avoidance beliefs and pain severity.

**Table 4:** Low-back pain intensity at one-year follow-up among the entire study population and in the sensitivity analyses among female nurses for each of the three fear-avoidance baseline categories

Fear-avoidance	Population	N	Mean	Std
Normal FABW (0–19)	All	1569	1.75	2.18
Moderate FABW (20–29)		290	3.11	2.68
High FABW (30–42)		74	4.41	2.38
Normal FABW (0–19)	Nurses	943	1.84	2.24
Moderate FABW (20–29)		182	3.04	2.52
High FABW (30–42)		44	4.77	2.26

## Discussion

This prospective cohort study demonstrates a notable association between FABW and the likelihood of increased LBP intensity and prolonged LBP duration among healthcare workers. The findings reveal that moderate and high FABW levels at baseline amplify the likelihood of increased LBP intensity and duration at one-year follow-up. These observations are consistent across the cohort, with sensitivity analysis in the subgroup of female nurses further highlighting the relevance of FABW in the context of LBP. Recognizing the importance of FABW offers crucial insights into the potential for targeted interventions to mitigate LBP among healthcare professionals.

Our findings align with the systematic review by Wertli et al. (2014b), which demonstrated FABW as a crucial prognostic factor for LBP, specifically as a predictor for work-related outcomes in subacute LBP cases [22]. This review, integrating outcomes from four cohort studies of patients with nonspecific LBP, identified a heightened risk of work-related consequences, including increased absenteeism, for individuals exhibiting high levels of FABW. Complementing this, our research finds support in studies conducted among diverse occupational groups, including a notable study among nurses in Japan, which documented a significant link between high fear-avoidance behavior about physical activity (FABP) and chronic, disabling LBP [25]. Furthermore, the role of psychosocial factors in the recovery and return-to-work process highlights the potential utility of the FABW subscale as a screening tool for identifying at-risk individuals [30].

In line with these perspectives, understanding the origin and development of FABW is critical for crafting effective interventions for LBP management. While FABW can be shaped by numerous elements, chronic musculoskeletal pain emerges as a fundamental trigger, leading to a cycle of fear of movement, work avoidance, and increased absenteeism [35]. This cycle seems to be exacerbated by a range of psychosocial workplace factors, and lifestyle choices, all contributing to the proliferation of FAB [23]. The cognitive interpretation of pain as a threat encourages avoidance behaviors, particularly reducing physical activity and potentially quality of care [36], a situation compounded by lower education and fitness levels that are linked to increased FAB and diminished exercise adherence among healthcare workers [37].

The comparison of odds ratios between the minimally and fully adjusted models reveals a consistent trend: the association between different levels of FABW and the likelihood of increased pain intensity and duration remains significant, albeit with a slight average reduction in the fully adjusted models. This subtle decrease suggests that while the influence of FABW on pain outcomes is predominant, it is slightly moderated by additional covariates such as education, BMI, smoking status, work experience, leisure activity, patient handling frequency, and psychosocial workplace factors. Despite this moderation, the fundamental relationship between FABW categories (Moderate vs Low, High vs Low) and pain outcomes consistently supports the intrinsic impact of fear-avoidance beliefs on pain development. This persistent influence underscores the critical role of FABW in both pain intensity and duration, emphasizing the importance of interventions focused specifically on addressing these beliefs.

The similarity in odds ratios between the minimally and fully adjusted models illustrates a consistent pattern: the risk associated with different levels of FABW regarding the likelihood of increased pain intensity and duration remains relatively stable, irrespective of the depth of adjustment for additional covariates, albeit with a minor average reduction in the fully adjusted models. This stability suggests that the influence of FABW on pain outcomes is not largely mediated by the included covariates such as education, BMI, smoking status, work experience, leisure activity, patient handling frequency, and psychosocial workplace factors. Moreover, the essential relationship between FABW categories (Moderate vs. Low, High vs Low) and pain outcomes also appears fundamentally consistent across the models, hinting at the intrinsic impact of fear-avoidance beliefs on pain development. These findings emphasize the critical role of FABW as a primary factor in both pain intensity and duration, reinforcing the need for targeted interventions focused on addressing these beliefs directly.

Our findings on the intensity of low-back pain at the one-year follow-up further supports this connection, showcasing how higher levels of FABW at baseline correlate with increased pain at one-year follow-up, particularly noted within the ‘High FABW’ category for both the general study population and the subgroup of female nurses. This direct link between elevated FABW and more severe pain outcomes aligns with previous research, emphasizing the transition from psychological fear-avoidance mechanisms to concrete, exacerbated pain experiences [24]. Such evidence firmly advocates for the implementation of targeted interventions aimed at addressing FABW from the outset, highlighting the importance of psychological factors in the comprehensive management and prevention of chronic LBP.

Given these insights, interventions targeting FAB are crucial in LBP management. Wertli et al. found that high FAB is linked to poor outcomes in short-term LBP cases, highlighting the importance of early, FAB-focused interventions [28]. Addressing FAB in treatments leads to better patient outcomes [28]. Psychological interventions, especially cognitive-behavioral therapy and psychoeducation, have proven effective for chronic LBP [38]. Additionally, physical methods like graded exposure therapy and workplace exercises have shown promising results. Our research group have previously demonstrated that workplace physical and cognitive-mindfulness training significantly reduced work-related FAB and LBP [39], and workplace exercises were more effective than home exercises in lowering LBP and FAB among healthcare workers [40, 41]. This evidence underscores the significance of incorporating FAB management in treatment protocols for healthcare professionals, given their physically and psychologically demanding roles. Addressing FAB can help maintain their wellbeing, productivity and quality of patient care, thereby preventing the adverse effects of chronic LBP.

### Strengths and Limitations

A significant strength of this study is the adjustment for baseline LBP and other confounding factors related to pain intensity and development of pain. Despite these adjustments having minimal impact on the odds ratios, they crucially account for the potential bias introduced by pre-existing pain conditions. This adjustment effectively addresses the potential influence of pre-existing pain on the connection between fear-avoidance beliefs and heightened LBP. However, certain limitations must be acknowledged. Firstly, the findings may not be universally applicable to other occupational groups, thus restricting the generalizability of outcomes beyond the specific population under investigation. However, it is plausible that the results apply to other job groups with physically demanding work tasks. Secondly, the utilization of self-reported data introduces the possibility of recall bias and subjective interpretation, potentially leading to underreporting of musculoskeletal disorders. Such limitations are inherent to questionnaire-based surveys [42]. The homogeneity of our study population, comprised solely of healthcare workers, acts as a significant strength. This focus minimizes extraneous variability and enhances the validity of our results concerning the impact of FABW on LBP outcomes. Moreover, our analysis, drawing from 1933 responses out of 7025 approached healthcare workers, faces potential selection bias. Participants with a prior interest or experience in LBP might skew our findings towards those more directly impacted by these issues, affecting the generalizability of our results. This necessitates a cautious interpretation of our study, recognizing it may not fully represent the broader healthcare worker population. Future studies should strive for more inclusive sampling to validate our findings across the healthcare sector.

Longitudinal studies with larger sample sizes and diverse populations are warranted to strengthen the generalizability of the results. Moreover, investigating the effectiveness of different interventions specifically targeting fear-avoidance beliefs will provide valuable insights into the most effective strategies for reducing fear-avoidance beliefs and improving LBP outcomes. Additionally, incorporating objective measures of physical activity and clinical assessments would enhance the accuracy and reliability of the findings as well as increase the understanding of the underlying mechanism of fear-avoidance behavior. Indeed, exploring the underlying mechanisms and pathways through which fear-avoidance beliefs influence LBP could uncover new therapeutic targets and inform the development of innovative interventions.

## Conclusion

In conclusion, our study links FABW to higher odds of intensified LBP intensity and prolonged duration. These findings underscore the critical role of addressing FABW in the development of effective pain management strategies, emphasizing the need for interventions tailored to mitigate these beliefs. The evidence provided lays a foundation for future research to explore effective interventions targeting FABW, aiming to reduce the prevalence and impact of LBP in healthcare settings. Our findings suggests that integrating psychological and behavioral approaches into LBP management protocols can enhance healthcare workers’ well-being and professional efficacy.

## Data Availability

The datasets generated during and/or analyzed during the current study are available from the corresponding author on reasonable request.
